# Physiological Effects of Conium Maculatum

**Published:** 1845-10-01

**Authors:** 


*Physiological effects of Conium Maculatum*—In the July No., of the American Journal of Med. Sciences, Pliny Earle M. D., Physician to Bloomingdale Asylum for the insane, N. Y., details some experiments upon his own person to determine the effects of Conium, and the quantity necessary to be taken to secure its specific effects.

He commenced with an extract prepared by Lee and Butler of Hartford Conn., in doses of i grain, and gradually increased the dose, daily, up to sixty grains, which he repeated for seven successive days. The effects of the medicine were slight vertigo and sense of fulness in the head, when 25 grains were taken morning, noon, and evening; increase of vertigo, and feeling as if the eyes were swollen and protuberant after 30 grs; weariness and weakness in the knees, were added after 45 grs; dimness of vision, a peculiar feeling of uneasiness and weakness in the *biceps brachii* muscle, and some dilation of pupils after 50 grs; increase of all the preceeding symptoms, with the addition of double vision after 60 grs. A sensation of warmth in the epigastrium was also perceived after taking it in large doses. It does not appear that any tendency to somnolency was produced.

A second series of experiments was subsequently made with an extract shortly before inported, bearing the mark of Mander, Weaver & Co. No sensible effects were produced until sixteen grains *terrin die* were taken. Sixty grains at morning, and seventy at noon and night, of the same day, produced similar effects, and to the same degree, as 35 grains of the American extract. Eighty grains in the morning, ninety at noon and one hundred at night, produced, in addition to the usual symptoms, a cerebral oppression which was extremely unpleasant. He had, however, cephalalgia before taking the medicine on that day. The pulse was reduced two or three beats below its natural frequency. There was no perceptible augmentation or diminution of the urine during these experiments.

From these results we must conclude, either that the extracts used were defective, or, that the potency of this medicine has been overrated. It seems to us that the former supposition is most probable. The question is one of considerable practical importance. It were to be wished that Dr. Earle had experimented with a number of different articles, both American and English; or, that those used, had been previously subjected to chemical analysis- The profession are under much obligation to him for these observations.

